# Supplementary material for: Advances in the Mechanistic Understanding of Iron Oxide Nanoparticles’ Radiosensitizing Properties
Source: Nanomaterials (Basel). 2023 Jan 2;13(1):201. doi: 10.3390/nano13010201 (PMC9823929; doi:10.3390/nano13010201)
Supplement: Supplementary file 1 [file nanomaterials-13-00201-s001.zip › nanomaterials-2082492-supplementary.pdf]

Supporting information

Advances in the Mechanistic Understanding of Iron Oxide Nanoparticles' Radiosensitizing Properties

Indiana Ternad[1], Sebastien Penninckx[2], Valentin Lecomte[1], Thomas Vangijzegem[1], Louise Conrard[3], Stéphane Lucas[4], Anne-Catherine Heuskin[4], Carine Michiels[4], Robert N. Muller[1,3], Dimitri Stanicki[1]*, Sophie Laurent[1,3]

Synthesis of iron oxide nanoparticles (IONPs)

Magnetic nanoparticles were synthesized by co-precipitation of iron salts in DEG according to a protocol previously described. A mixture of ferrous chloride tetrahydrate salt (45 mmol; 8.9 g) and ferric chloride (45%; 37 mmol; 9.1 ml) in DEG (250 ml) was heated at 170 °C under a nitrogen atmosphere and under stirring. After 15 min at that temperature, solid sodium hydroxide (15 g) was added in order to prevent any dilution. The solution was stirred for 1 h at 170 °C. After cooling, the magnetic particles were isolated by magnetic decantation (B_0 = 0.5 T), after which the black precipitate was washed five times with an aqueous solution of nitric acid (200 ml, 1 M). Finally, the particles were dispersed in deionized water, sonicated (45 minutes), and centrifuged (16,500 g; 45 min) to remove aggregates.

The IONP suspension (20 ml; [Fe] = 250 mM) was diluted with DMF (50 ml) and water was evaporated under reduced pressure. TEPSA (25 mmol; 7.1 ml) was then slowly added to the nanoparticle dispersion in DMF; water was then added (4.3 ml), followed by an aqueous solution of TMAOH (1 M; 2.5 mmol; 2.5 ml) at room temperature and under stirring. The solution was heated to 100 °C for 24 h under continuous stirring. The magnetic nano-objects were collected after pouring the suspension in an acetone–diethylether mixture and magnetic decantation. After washing with acetone, the black precipitate was dispersed in water and purified by membrane filtration (membrane cut-off: 30 kDa) and finally centrifuged (16,500 g; 45 minutes). An amount of 3.4 COOH/nm^2 has been estimated from TGA analysis.

Synthesis of rhodamine-tagged IONPs

To obtain rhodamine-labelled IONPs, a small amount of aminated-rhodamine [33] (TFA salt ; 1 µmol; 0.8 mg) was added to an aqueous dispersion of TEPSA-modified nanoparticles ([Fe] =

100 mM ; 2 ml) in the presence of *N*-(3-dimethylaminopropyl)-*N′*-ethylcarbodiimide hydrochloride (50 µmol; 10 mg) as a coupling agent. After one night under stirring at pH 7.5, the suspension was purified by membrane filtration (membrane cut-off = 30 kDa), then centrifuged (16 500 g; 40 minutes).

Synthesis of PEGylated IONPs

To obtain PEGylated IONPs, the previously synthesized carboxylated-IONPs (5 mL ; [Fe] = 200 mM) was treated with *O*-(2-aminoethyl)-*O′*-methylpolyethyleneglycol (MW_{PEG} = 5000 g/mol ; 0.15 mmol ; 750 mg) and EDC (0.25 mmol ; 48 mg). The pH was then adjusted to 6.5 and the mixture was stirred at room temperature. After 15 hours of reaction, the suspension was purified by dialysis (membrane cut-off: 12-14kDa), followed by membrane filtration (membrane cut-off = 30 kDa).

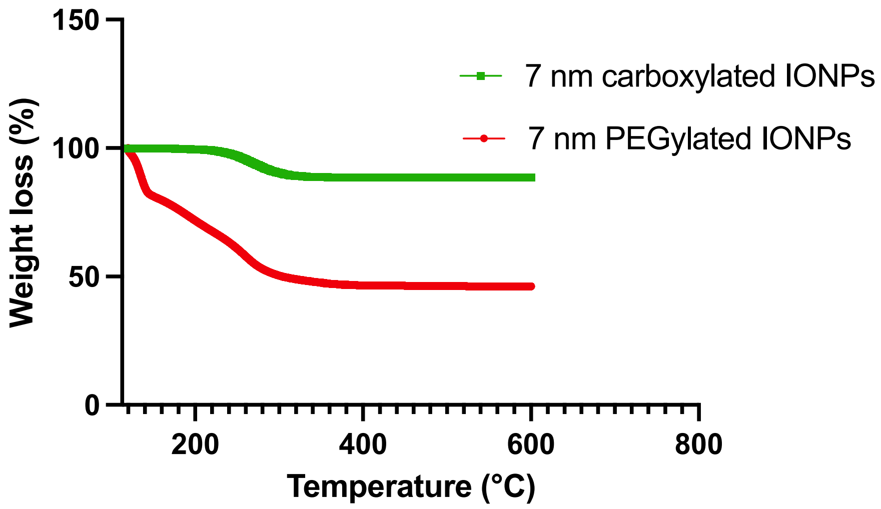

Fig. S1 Thermogravimetric analysis of 7 nm carboxylated IONPs (green curve) and 7 nm IONPs PEG_{5000} (red curve)

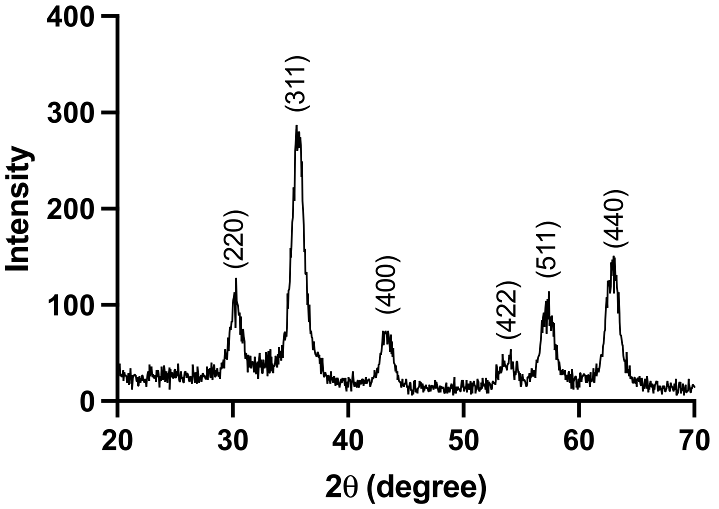

Fig. S2 XRD diffraction patterns of IONPs

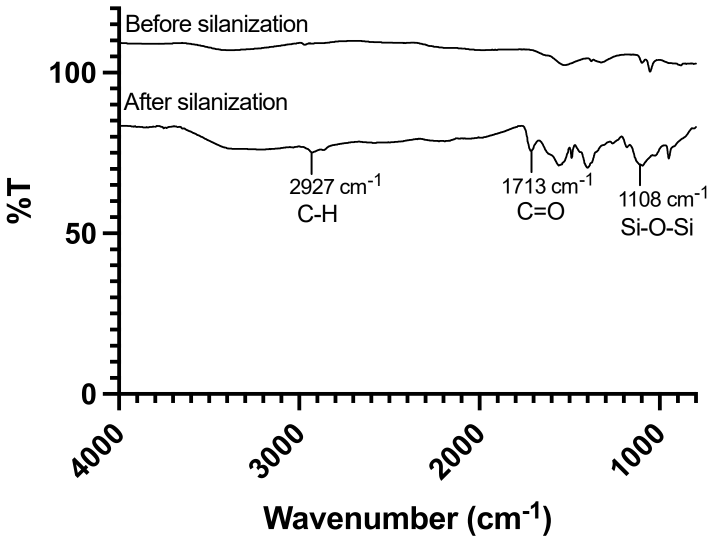

Fig. S3 Comparison of the FTIR spectra obtained for naked particles and carboxylated IONPs

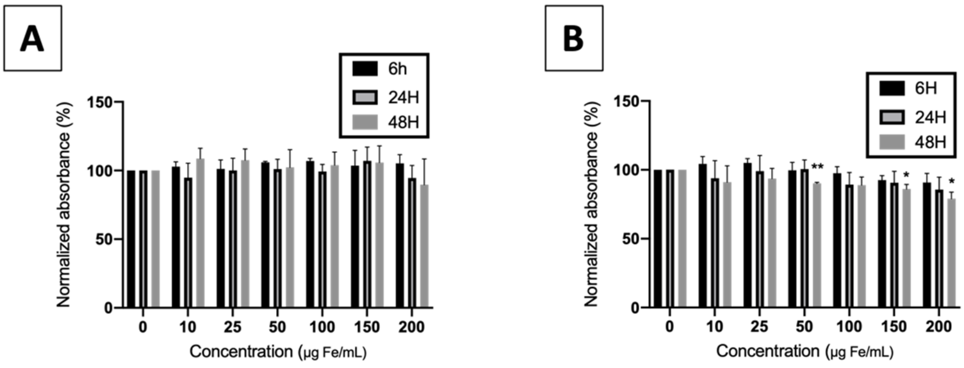

Fig. S4 Cell viability of A549 cells incubated for 6h/ 24 h/ 48h with different concentrations of (A) 7 nm carboxylated IONPs and (B) 7 nm IONPs PEG_{5000} and assessed using a MTT assay. Mean values of absorbance for treated cells normalized by mean values of untreated cells of three independent experiments. The multiple comparisons to the control were performed using one-way ANOVA Dunnett's test.

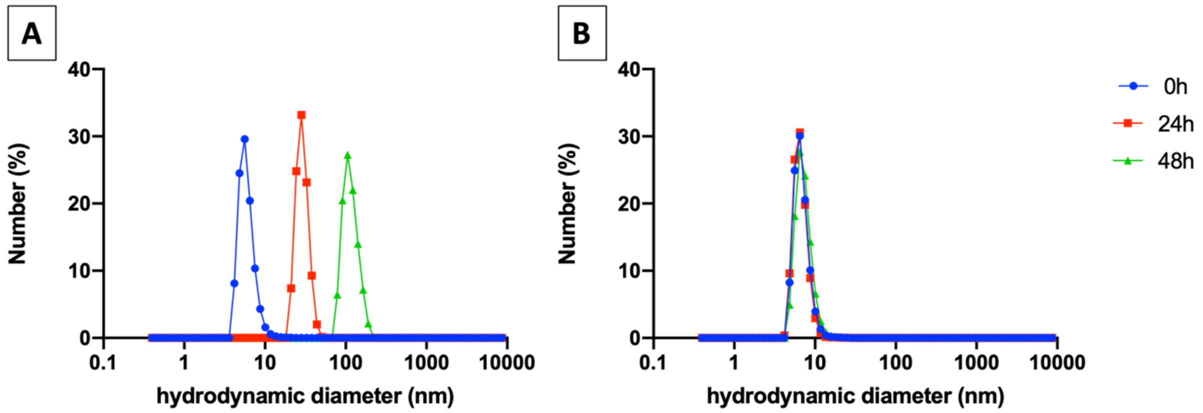

Fig. S5 Evolution of the hydrodynamic diameter of (A) 7 nm carboxylated IONPs (B) 7 nm IONPs PEG_{5000} at 50 µg of Fe/mL in culture media (MEM) over time.

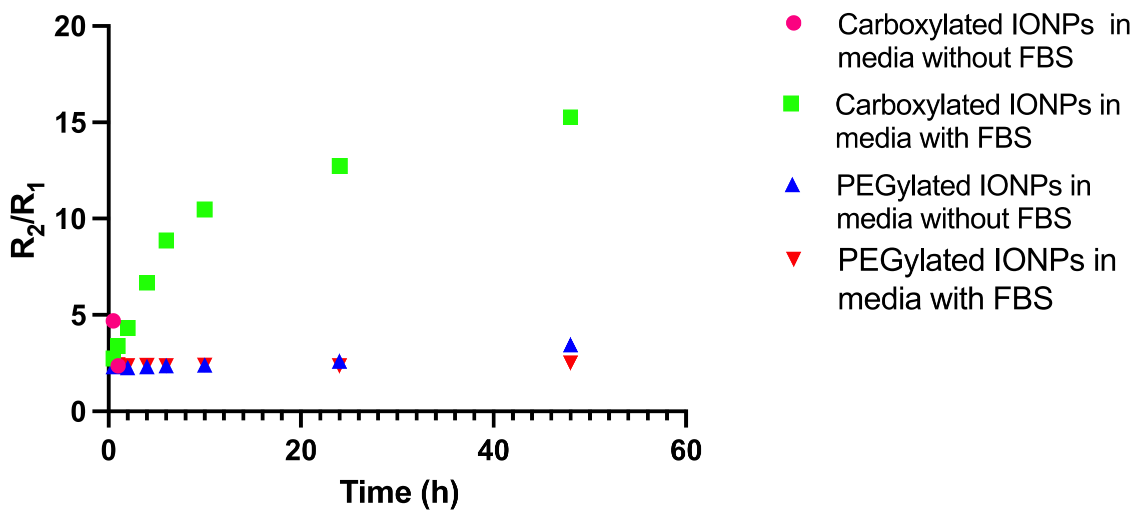

Fig. S6 Evolution of R_2/R_1 ratio of 7 nm carboxylated IONPs and 7 nm IONPs PEG_{5000} at 50 µg of Fe/mL in media with and without FBS, over time.

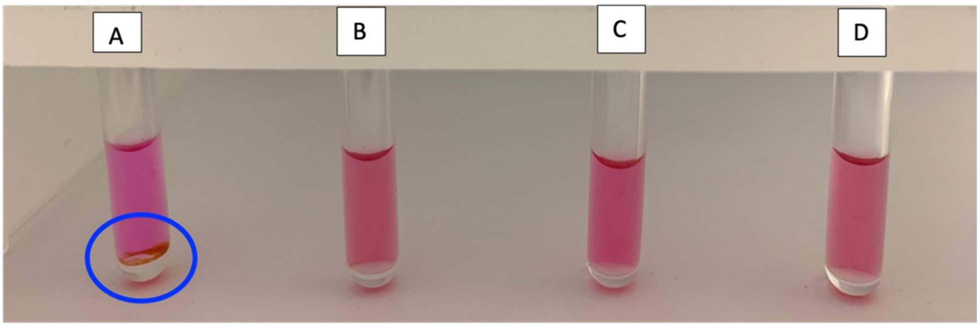

FBS	–	+	–	+
7 nm carboxylated IONPs	+	+	–	–
7 nm IONPs PEG_{5000}	–	–	+	+

Fig. S7 Evolution of the stability of 7 nm carboxylated IONPs and 7 nm IONPs PEG_{5000} at 50 µg of Fe/mL in culture media without FBS (A, C) / with FBS (B,D) after 24h.

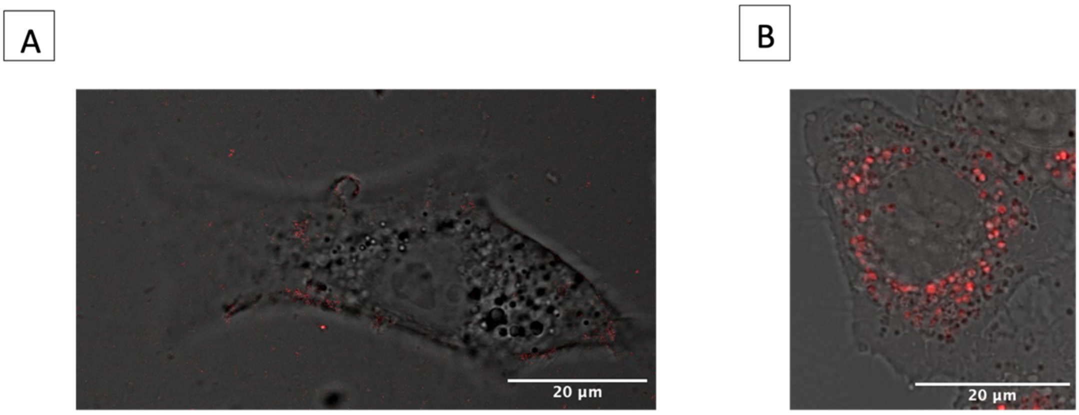

Fig. S8 Confocal microscopy image showing cellular uptake of Rhodamine labelled 7 nm carboxylated IONPs by A549 cells after A) 2h B) 48h of incubation. Merged superposition of a single projection of red channel- Rhodamine-IONPs and grey channel- DIC.

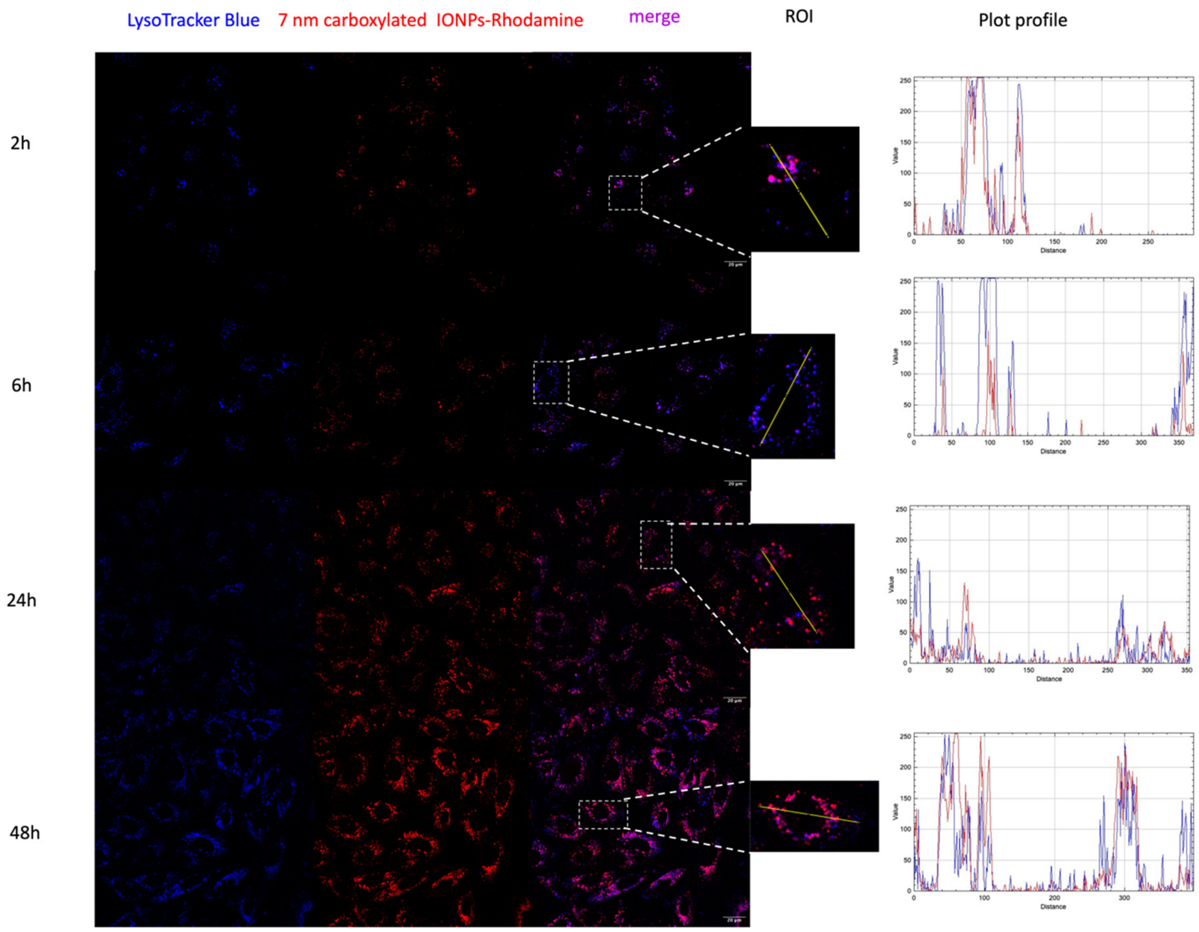

Fig. S9 Confocal microscopy micrographs of iron oxide nanoparticle uptake in A549 cells and intracellular localization. Cells preincubated with 50 µg of Fe / ml of 7 nm carboxylated IONPs for 2h; 6h; 24h; 48h (scale bar: 20 µm). Red channel- Rhodamine-IONPs and Blue channel- LysoTracker Blue**.** Intensity profiles of 7 nm carboxylated IONPs (red) and lysotracker (blue) were obtained using Fiji software along a straight line through a representative cell to assess colocalization.

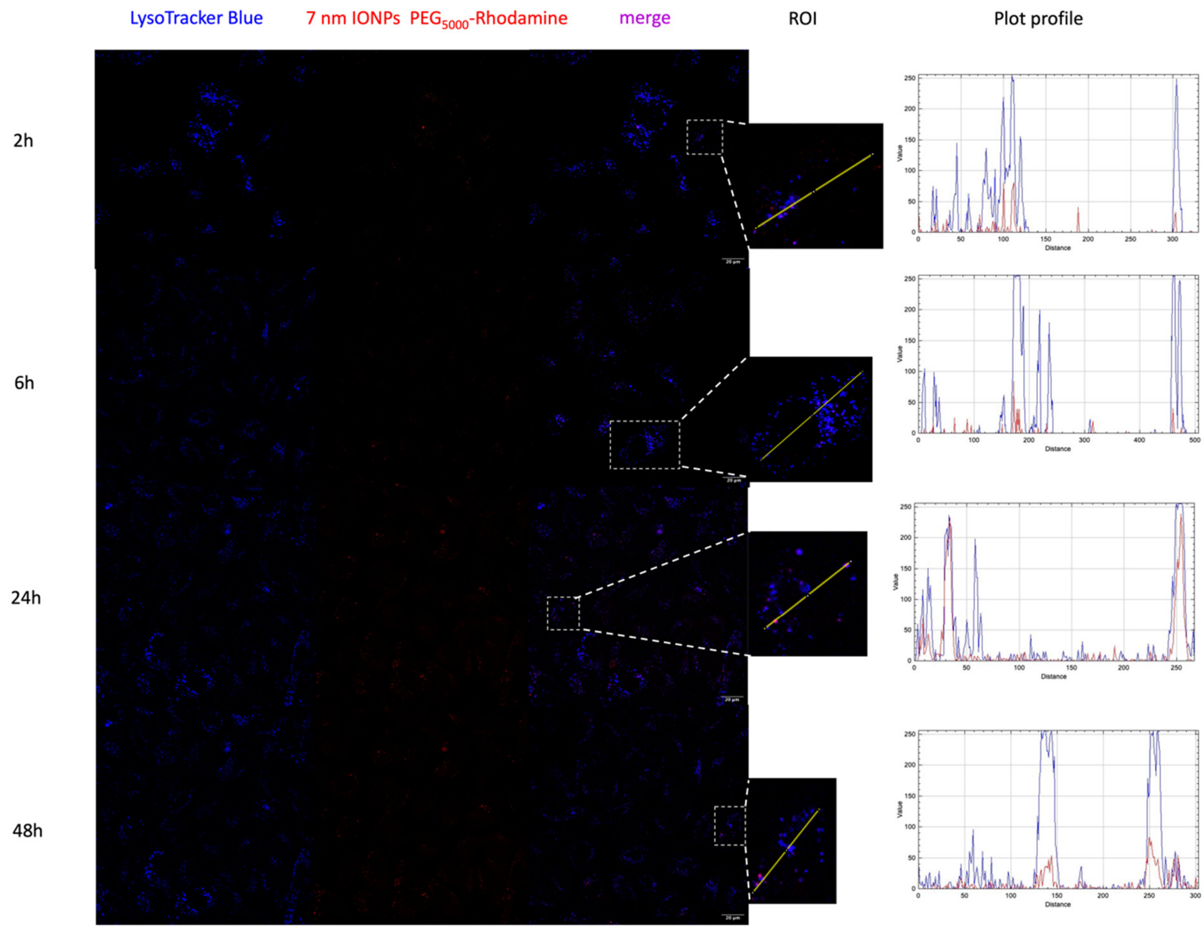

Fig. S10 Confocal microscopy micrographs of iron oxide nanoparticle uptake in A549 cells and intracellular localization. Cells preincubated with 50 µg of Fe/ml of 7 nm IONPs PEG_{5000}-Rhodamine for 2h; 6h; 24h; 48h (scale bar: 20 µm). Red channel- Rhodamine-IONPs and Blue channel- LysoTracker Blue**.** Intensity profiles of 7 nm IONPs PEG_{5000} (red) and lysotracker (blue) were obtained using Fiji software along a straight line through a representative cell to assess colocalization.

Table S1: Colocalization percentage of rhodamine-labeled 7 nm carboxylated IONPs or 7 nm IONPs PEG_{5000} with lysosomes in A549 cells

	**7 nm carboxylated IONPs (n)**	**7 nm IONPs PEG_{5000} (n)**
2h	91±8 (10)	91±9 (9)
6h	88±8 (11)	96±6 (9)
24h	75±15(11)	89 ± 15 (11)
48h	91±8 (15)	96 ± 3 (12)

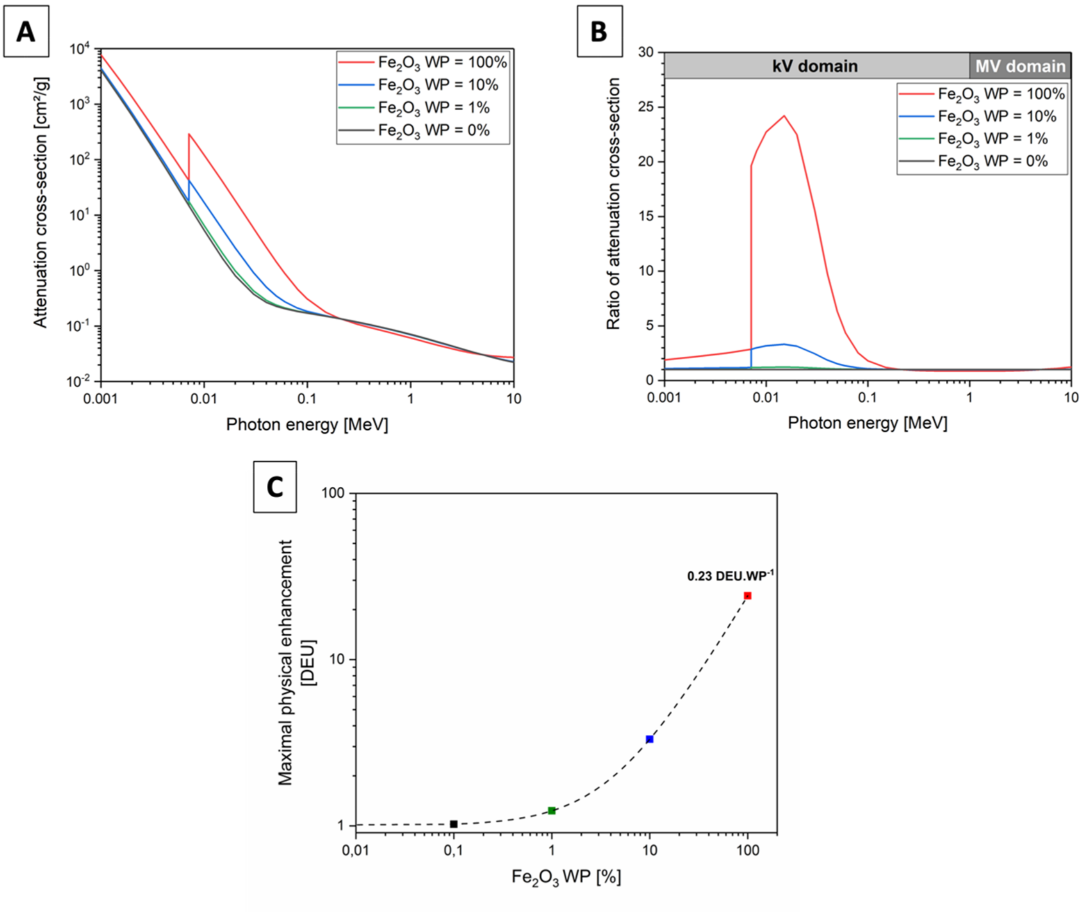

Fig. S11 (A) Attenuation cross-section of photons in water (black) and Fe_2O_3 as a function of photon energy. (B) Ratio of Fe_2O_3 attenuation cross-section on water attenuation cross-section depending on photon energy. (C) Maximal dose enhancement for photons (calculated for an energy of 15 keV depending on the Fe_2O_3 weight percent (WP). An enhancement of 1 DEU (dose enhancement unit) means that the delivered dose doubles

when Fe_2O_3 nanoparticles are present in the medium. Photon data were derived from the NIST XCOM database.

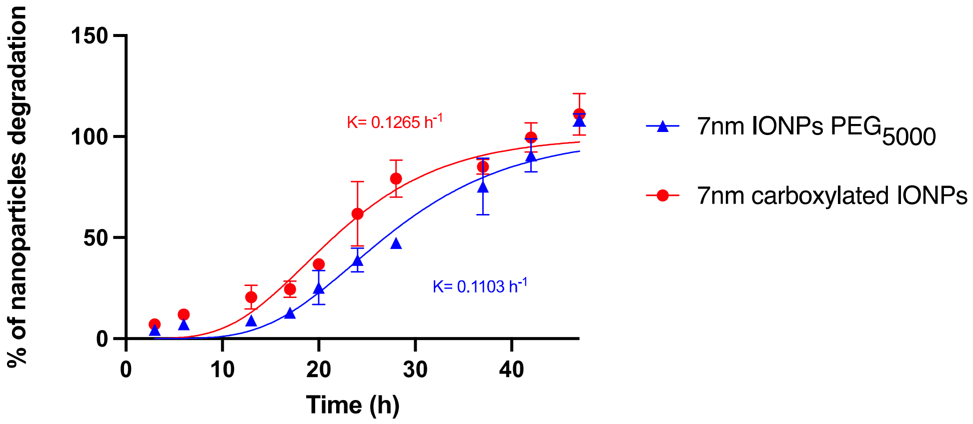

Fig.S12 Degradation of 7 nm IONPs PEG_{5000} and 7 nm carboxylated IONPs in an artificial lysosomal fluid over time. The percentage of iron ions released was measured by the Prussian blue method. Data are plotted as mean ± SD from 3 independents experiments.

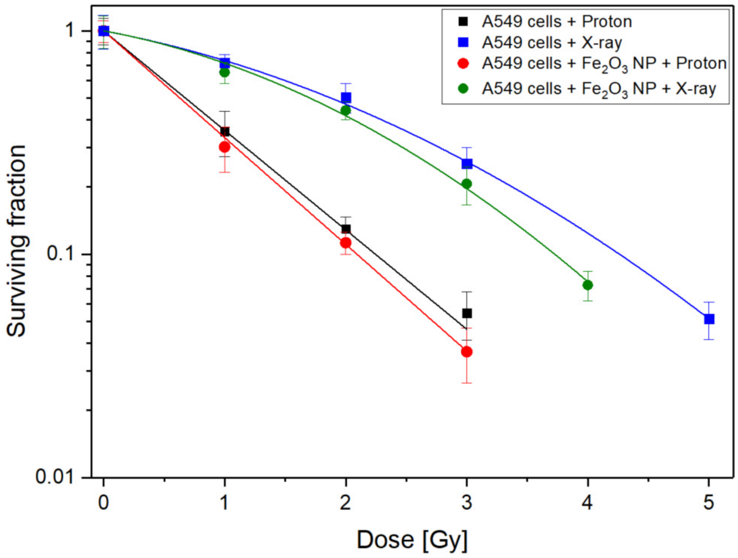

Fig.S13 Clonogenic survival fraction curve fitted by linear quadratic model for A549 cells incubated with and without 7 nm IONPs PEG_{5000} (50 µg of Fe/mL) for 24h, irradiated with X-rays (blue, green curves) or irradiated with protons (black, red curves)

Table S2: Calculated α, β coefficients and sensitization enhancement ratio at 10% survival for A549 cells irradiated by a 25 keV/µm proton beam or 225 kV x-rays after being pre-

incubated during 24 h with 50 µg Fe/mL of 7 nm IONPs PEG_{5000} compared to in untreated control cells (without IONPs). Data were calculated using linear-quadric model on data from Fig. S12

Beam	Sample	α [Gy^{-1}]	β [Gy^{-2}]	α/β [Gy]	$SER_{10\%}$
**X-ray**	A549	0.23 ± 0.03	0.07 ± 0.01	3.3	-
	A549 + IONPs	0.32 ± 0.05	0.07 ± 0.02	4.6	1.10 ± 0.04
**Proton**	A549	1.02 ± 0.01	0	-	-
	A549 + IONPs	1.10 ± 0.01	0	-	1.08 ± 0.02
